# Patient-specific Ti-6Al-4V lattice implants for critical-sized, weightbearing limb reconstruction: average 45-month surgical, oncological, and functional follow-up

**DOI:** 10.1302/2633-1462.74.BJO-2026-0026.R1

**Published:** 2026-04-10

**Authors:** Amit Benady, Netanel Sharabi, Eran Golden, Ortal Segal, Omri Merose, Amir Sternheim, Solomon Dadia, Yair Gortzak

**Affiliations:** 1 Levin Center for 3D printing and Surgical Innovation, Tel Aviv Sourasky Medical Center, Tel Aviv-Yafo, Israel; 2 The Department of Orthopedic Surgery, Tel Aviv Sourasky Medical Center, Tel Aviv-Yafo, Israel; 3 Department of Anatomy and Anthropology, Grey school of medicine, Tel Aviv University, Tel Aviv-Yafo, Israel; 4 The National Unit of Orthopedic Oncology, Tel Aviv Sourasky Medical Center, Tel Aviv-Yafo, Israel

**Keywords:** Ti-6Al-4V lattice implants, Limb salvage, Patient-specific reconstruction, Additive manufacturing, Critical-sized bone defects, bone tumour resections, limb salvage surgery, Osteotomies, Musculoskeletal Tumor Society (MSTS) scores, limb reconstruction, deep infections, nonunion, revision surgeries, tibia, Functional outcomes

## Abstract

**Aims:**

Patient-specific Ti-6Al-4V lattice implants present a new era for reconstruction of weightbearing segmental defects; however, robust clinical data from larger cohorts with longer follow-up remain limited. Building on our previous study that detailed the engineering, design, and surgical workflow of these implants, this study focuses on clinical outcomes. Specifically, we aimed to: 1) characterize surgical complications and limb-salvage rates; 2) report resection margins and oncological status at the latest follow-up; and 3) describe functional outcomes.

**Methods:**

This retrospective single-centre study includes 29 patients treated between January 2016 and December 2024 with long-bone tumour resection (n = 28) or post-traumatic nonunion (n = 1) of the femur or tibia. Resections were guided by intraoperative 3D-printed osteotomy patient-specific instruments followed by reconstruction with customized Ti-6Al-4V lattice implants. Minimum follow-up was 12 months. Surgical complications, reoperations, resection margins, metastasis, and local recurrence were recorded. Musculoskeletal Tumor Society (MSTS) scores were obtained at latest follow-up.

**Results:**

The mean age was 26.2 years (SD 18.0); anatomical sites were tibia (15/29, 52%) and femur (14/29, 48%). Early complications occurred in 3/29 (10.3%; one haematoma, two deep infections); late complications occurred in 4/29 (13.7%; one deep infection requiring staged revision with implant removal and fibular grafting, one mechanical failure revised to a megaprosthesis at four months, one subtalar fusion for symptomatic nonunion, and one component loosening and subluxation). Limb salvage was achieved in 27/29 patients (93.1%). At latest follow-up, 23 patients were with no evidence of disease, two alive with evidence of disease, and three dead of disease; metastasis occurred in seven patients and local recurrence in four patients. The median MSTS at latest follow-up was 80% (IQR 60% to 87%).

**Conclusion:**

In this heterogeneous cohort, patient-specific Ti-6Al-4V lattice implants achieved reliable reconstruction with acceptable complication rates, high limb salvage, and oncological control, with high functional outcomes, supporting this approach as a practical and effective solution for critical-sized, weightbearing defects reconstruction.

Cite this article: *Bone Jt Open* 2026;7(4):507–518.

## Introduction

Limb salvage has become the gold standard for the surgical treatment of lower-limb bone sarcomas, owing to advances in operative precision enabled by adjuvant therapy protocols, high-resolution imaging, preoperative simulation, and intraoperative patient-specific instruments (PSIs).^1,2^ Nonetheless, two fundamental challenges persist: 1) the extensive segmental bone loss created by oncological resection, which frequently results in critical-sized defects, typically defined in long bones as osseous discontinuities exceeding 3 cm that lack intrinsic capacity for spontaneous healing;^3^ and 2) tumour resections performed adjacent to native joints or other complex anatomical structures, which constrain margins and reconstruction options.^2,4^ The principal objective of reconstruction in such cases is the restoration of structural integrity and limb function while minimizing the risk of complications that may necessitate revision surgery.^5^ The most widely used reconstructive approach involves biological grafting, particularly structural allografts with or without adjunctive vascularized fibular autografts.^6-9^ These grafts facilitate osseous regeneration via osteoinductive, osteoconductive, and osteogenic pathways, provided that sufficient biological integration is achieved.^10^ However, biological reconstruction is frequently associated with high complication rates, including graft fracture, nonunion, delayed union, and deep infection. Additive manufacturing (AM) has emerged as a transformative technology in orthopaedic surgery.^1,2,4,10-12^

The rapid evolution of AM has enabled fabrication of metallic implants with complex geometries, such as porous lattice architectures, unattainable using conventional manufacturing.^13^ These implants can be tailored to patient-specific anatomical data derived from CT or MRI imaging, enabling a personalized approach that enhances osseous regeneration.^14^ Lattice-based designs offer functional advantages, including tunable mechanical properties and improved potential for osseointegration.^15^ When optimized, these structures can achieve an elastic modulus approximating that of native bone, mitigating stress shielding, enhancing implant-host congruity, reducing periprosthetic bone resorption, and accelerating bone regeneration and integration at the bone–implant interface. Given these benefits, customized AM implants are gaining traction for reconstruction of segmental bone defects and complex anatomical sites and are increasingly regarded as a viable alternative to biological grafts.^16-18^ For load-bearing skeletal reconstruction, titanium-based alloys, particularly Ti-6Al-4V, are widely used due to their low density, high mechanical strength, corrosion resistance, and proven biocompatibility.^19^ The ability to custom-design Ti-6Al-4V implants for anatomically complex defects marks a significant advancement in orthopaedic reconstructive strategies.

This study builds upon our previous publication,^15^ which detailed the engineering aspects and surgical workflow of patient-specific Ti-6Al-4V additive-manufactured implants for critical-sized, weightbearing bone defects. That report included five representative patients emphasizing design and fabrication.

The current study shifts the focus towards clinical outcomes, reporting on a cohort of 29 patients treated with the same approach. To our knowledge, this represents the largest clinical series to date with the longest follow-up using this reconstructive method, providing a substantial contribution to the growing evidence base supporting additive manufacturing in orthopaedic surgery.

## Methods

The proposed workflow was carried out on 29 patients who underwent surgery between January 2016 and December 2024 at the National orthopaedic Oncology Unit at Tel Aviv Sourasky Medical Center. The inclusion criteria were primary bone sarcoma in a weightbearing bone, either the femur or tibia (apart from one case, which was nonunion due to trauma). All surgeries involved a long-bone tumour resection and reconstruction of critical-sized bone defects with a patient-specific Ti-6Al-4V implant. All patients were invited for regular follow-up visits to monitor local recurrence, metastases, and functional outcomes. Postoperative follow-up lasted for 12 to 111 months according to the standard schedule and strategy for conventional limb salvage surgery. Soft-tissue margins were classified as positive (R2), negative with microscopic residual disease (R1, < 1 mm), or negative with no residual disease (R0, ≥ 1 mm). Functional outcomes were assessed using the 1993 Musculoskeletal Tumor Society (MSTS-93) scoring system^20^ at the final follow-up and converted to percentages of the maximum score. Each patient received specific surgery approval from the Ministry of Health to use a customized Ti-6Al-4V implant (Sharon Tovia, Israel). This study was approved by the Research Ethics Committee of Tel Aviv Sourasky Medical centre.

### Image acquisition and processing

Cross-sectional imaging of the affected limb was performed using CT (SOMATOM X.cite; Healthineers Siemens, Germany) and MRI (MAGNETOM Vida; Healthineers Siemens) scans, typically about two weeks prior to the planned operation. High-resolution CT scans, with slice thicknesses between 0.5 mm and 1 mm, were taken to visualize the bone anatomy in detail, while MRI scans with 4 mm slices were used to define the tumour boundaries and surrounding soft-tissue structures. The resulting 2D CT and MRI datasets were then imported into specialized image processing software (Mimics; Materialise, Belgium), where segmentation and fusion enabled the creation of a 3D digital model that accurately represented both the skeletal framework and the extent of the tumour. Once segmentation was finished, the model was exported as a stereolithography (STL) file and imported into a computer-aided design (CAD) environment (3matic; Materialise).

### Anatomical model and patient-specific instrument design and 3D printing

**Fig. 1:**
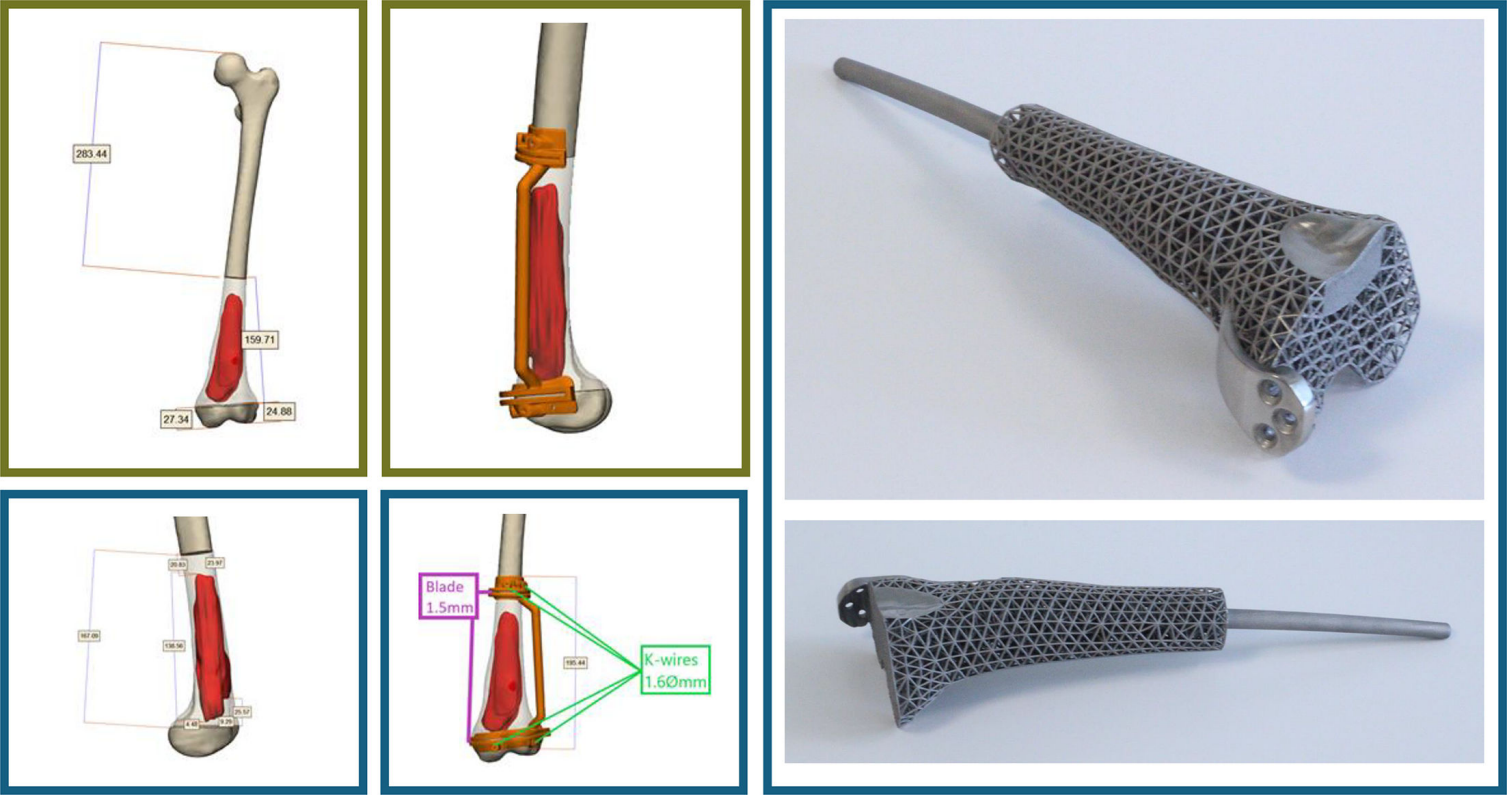
Preoperative 3D surgical planning for a 17-year-old female with a distal femoral osteosarcoma (patient #10). Virtual tumour segmentation and osteotomy planes were defined to achieve clear margins while preserving maximal bone stock. On the right, the corresponding patient-specific Ti-6Al-4V implant is shown.

**Fig. 2:**
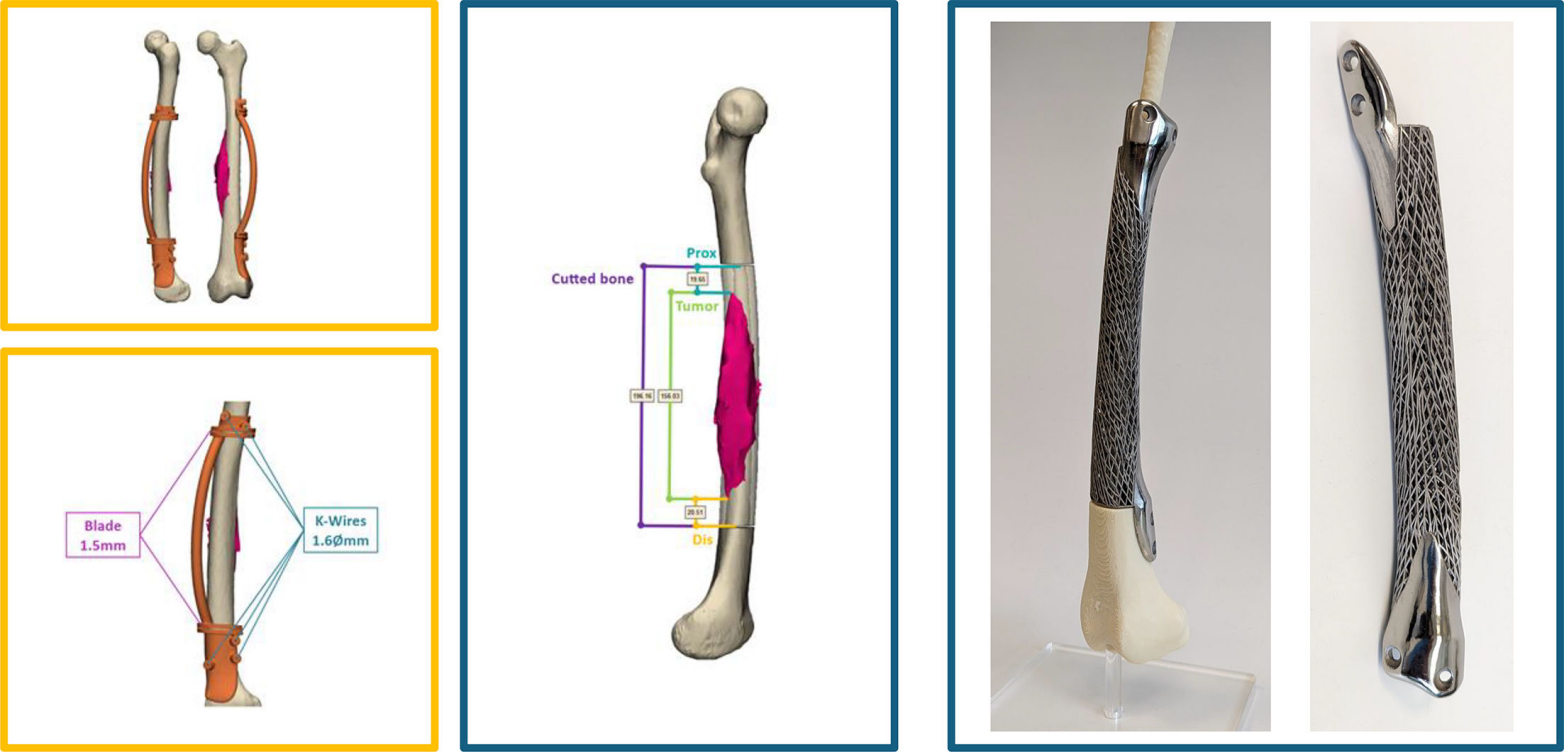
Preoperative 3D surgical planning for a 14-year-old female with a mid-shaft femoral Ewing's sarcoma (patient #12). The resection volume, osteotomy guides, and fixation plane were designed based on patient imaging. The corresponding patient-specific Ti-6Al-4V lattice implant is displayed on the right.

**Fig. 3:**
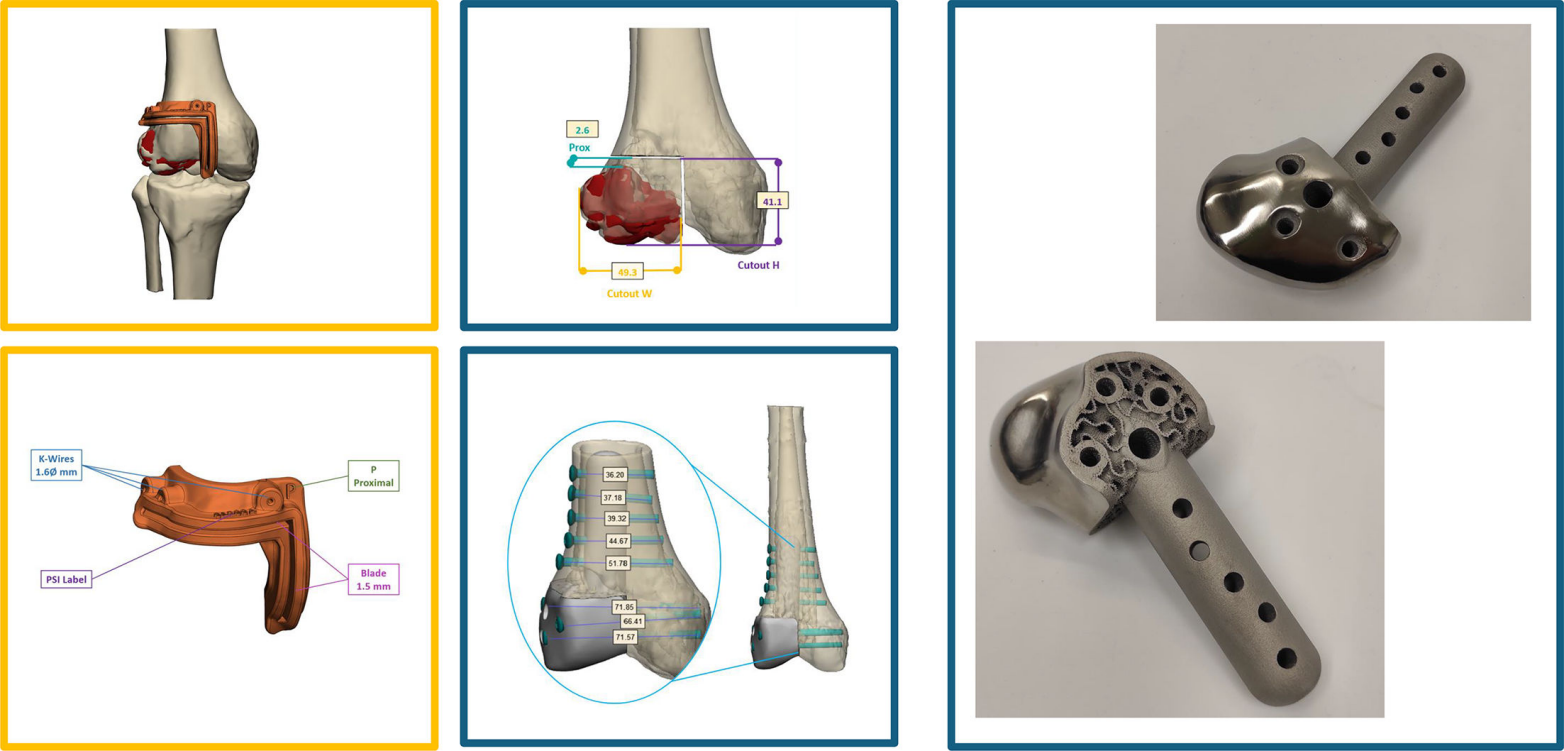
Preoperative 3D surgical planning for a 15-year-old male with distal femoral chondroblastoma (patient #19). Patient-specific osteotomy guides and fixation strategy were modeled to ensure accurate tumour resection and implant fit. On the right, the custom Ti-6Al-4V lattice implant is shown. PSI, patient-specific instrument.

**Fig. 4:**
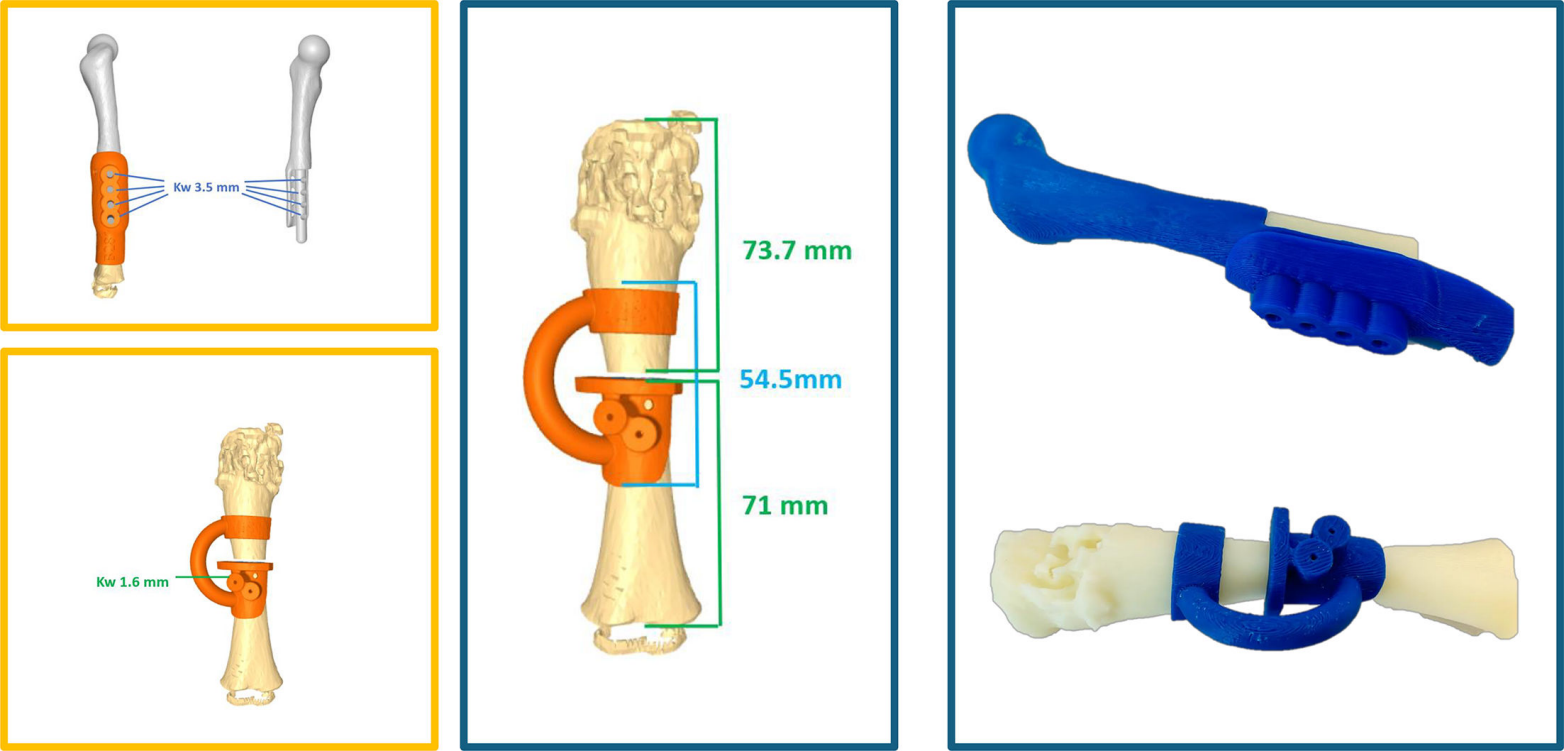
Preoperative 3D surgical planning for a two-year-old female with distal femoral osteosarcoma (patient #21). The tumour boundaries and resection planes were mapped using patient imaging data. On the right, a 1:1 anatomical model illustrates the planned resection and implant geometry.

The surgical approach was determined collaboratively by the surgeon and a medical engineer or designer (OS, OM, AS, SD, YG), tailored to each patient’s clinical presentation and pathology. Following multidisciplinary team approval of the surgical plan, PSIs were designed based on the required cutting planes, ensuring precise intraoperative guidance. Each PSI featured unique geometrical adaptations to match the patient’s bone morphology. Upon finalization of the surgical plan and digital model, full-scale anatomical models of the limb, including the tumour, and the PSI were 3D printed at a 1:1 ratio. Bone models were fabricated from acrylonitrile styrene acrylate (ASA) ivory filament (360 to 50240, Stratasys, Israel) on a Fortus 450mc fused deposition modelling (FDM) printer (Stratasys) at a resolution of 250 μm. For detailed, high-resolution tumour models, VERO family materials (RGD837; Stratasys) in multiple colours were printed using a PolyJet J750 printer (Stratasys) with a 16 μm resolution. PSIs were 3D-printed from certified ULTEM 1010 in various grades (355 to 02320; Stratasys) using the Fortus 450mc FDM printer at a 330 μm resolution. ULTEM 1010 was selected for its biocompatibility, mechanical strength, and thermal resistance, allowing for sterilization by high-temperature autoclaving required for intraoperative use. After fabrication, the PSIs were cleaned, double-packed, and underwent standard autoclave sterilization at 135°C. Figures 1 to 4 show the presurgical planning for four representative patients.

### Patient characteristics

A total of 29 patients (18 males, 11 females) underwent reconstruction of critical-sized, weightbearing bone defects using patient-specific 3D-printed titanium Ti-6Al-4V cages. The mean age at surgery was 26.2 years (SD 18.0; 2 to 68). The tibia was affected in 15/29 of cases (52%), and the femur in 14/29 (48%). Underlying diagnoses included osteosarcoma (n = 13), Ewing's sarcoma (n = 7), adamantinoma (n = 2), chondrosarcoma (n = 2), with single cases of malignant fibrous histiocytoma, desmoplastic fibroma, chondroblastoma, and nonunion. The median clinical follow-up was 33 months (12 to 111) (Table I).

**Table I.** Demographic details, diagnosis, and follow-up.

Patient #	Age at surgery, yrs	Sex	Pathology/diagnosis	Site	Follow-up, mnths
1	32	Male	Nonunion	Tibia	111
2	54	Male	Synovial sarcoma	Tibia	108
3	23	Male	Desmoplastic fibroma	Tibia	106
4	27	Male	Ewing’s sarcoma	Tibia	104
5	44	Male	MFH of bone	Tibia	98
6	21	Male	OSA	Tibia	67
7	8	Male	OSA	Femur	65
8	16	Male	Ewing’s sarcoma	Femur	59
9	20	Male	Ewing’s sarcoma	Tibia	50
10	17	Female	OSA	Femur	48
11	14	Male	Ewing’s sarcoma	Tibia	45
12	14	Female	Ewing’s sarcoma	Femur	43
13	35	Female	Adamantinoma	Tibia	37
14	14	Male	OSA	Femur	33
15	14	Female	OSA	Femur	34
16	18	Female	OSA	Tibia	33
17	68	Male	Adamantinoma	Tibia	32
18	7	Female	OSA	Femur	30
19	15	Male	Chondroblastoma	Femur	29
20	58	Female	OSA	Tibia	28
21	2	Female	OSA	Femur	28
22	12	Male	OSA	Femur	23
23	56	Male	Chondrosarcoma	Tibia	25
24	16	Female	Ewing’s sarcoma	Femur	19
25	33	Male	Ewing’s sarcoma	Tibia	20
26	14	Male	OSA	Femur	16
27	13	Female	OSA	Tibia	12
28	59	Male	Chondrosarcoma	Femur	13
29	37	Female	OSA	Femur	12

MFH, malignant fibrous histiocytoma; OSA, osteosarcoma.

### Ti-6Al-4V lattice implant design and fabrication

Each patient-specific implant consisted of a custom-designed titanium lattice scaffold integrated with orthopaedic fixation components. Two approaches were used: either the entire construct, including the lattice scaffold and fixation plates, was produced as a single, unified 3D-printed piece, or the lattice scaffold was printed with minimal integrated features and subsequently reinforced intraoperatively using off-the-shelf orthopaedic implants for additional fixation and stability. This dual strategy allowed for optimal adaptation to the patient’s anatomical requirements and surgical needs, while leveraging both advanced additive manufacturing and conventional orthopaedic hardware. Finite element analysis was performed with SimSolid software (Altair, USA) on the 3D model of the designed implants. The full technical specifications of the patient-specific Ti-6Al-4V lattice implant design and manufacturing process are detailed in our previous publication.^15^

### Surgical procedures and clinical follow-up

**Fig. 5:**
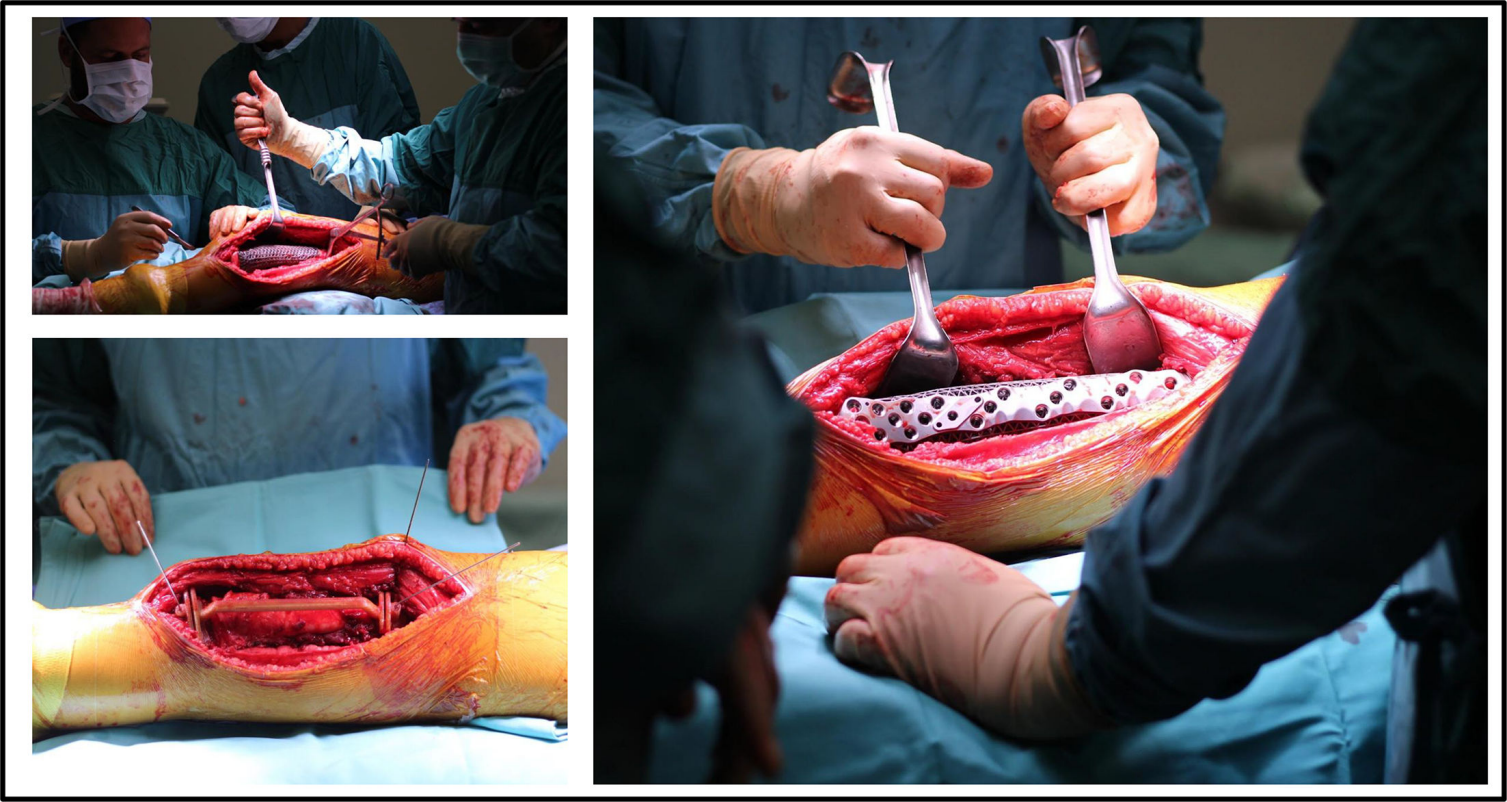
Intraoperative views of a 17-year-old female with distal femoral osteosarcoma (patient #10). Following tumour resection guided by patient-specific osteotomy instruments, the custom Ti-6Al-4V lattice implant was positioned within the defect to restore bone continuity and alignment before fixation.

**Fig. 6:**
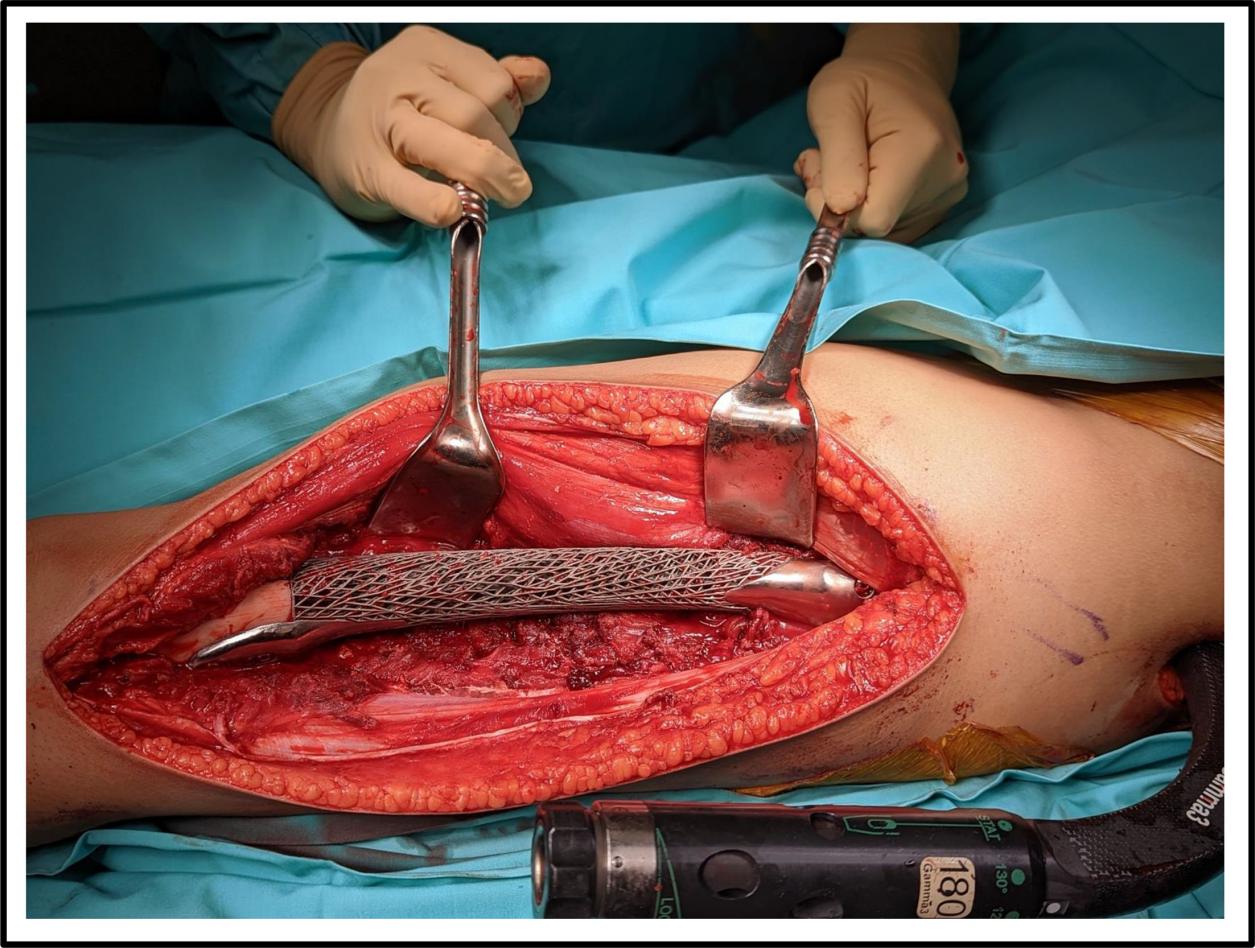
Intraoperative view of a 14-year-old female with distal femoral Ewing's sarcoma (patient #12). The resected segment was reconstructed using a patient-specific Ti-6Al-4V lattice implant designed from pre-operative 3D planning. The implant demonstrates precise fit and anatomical conformity within the defect prior to final fixation.

**Fig. 7:**
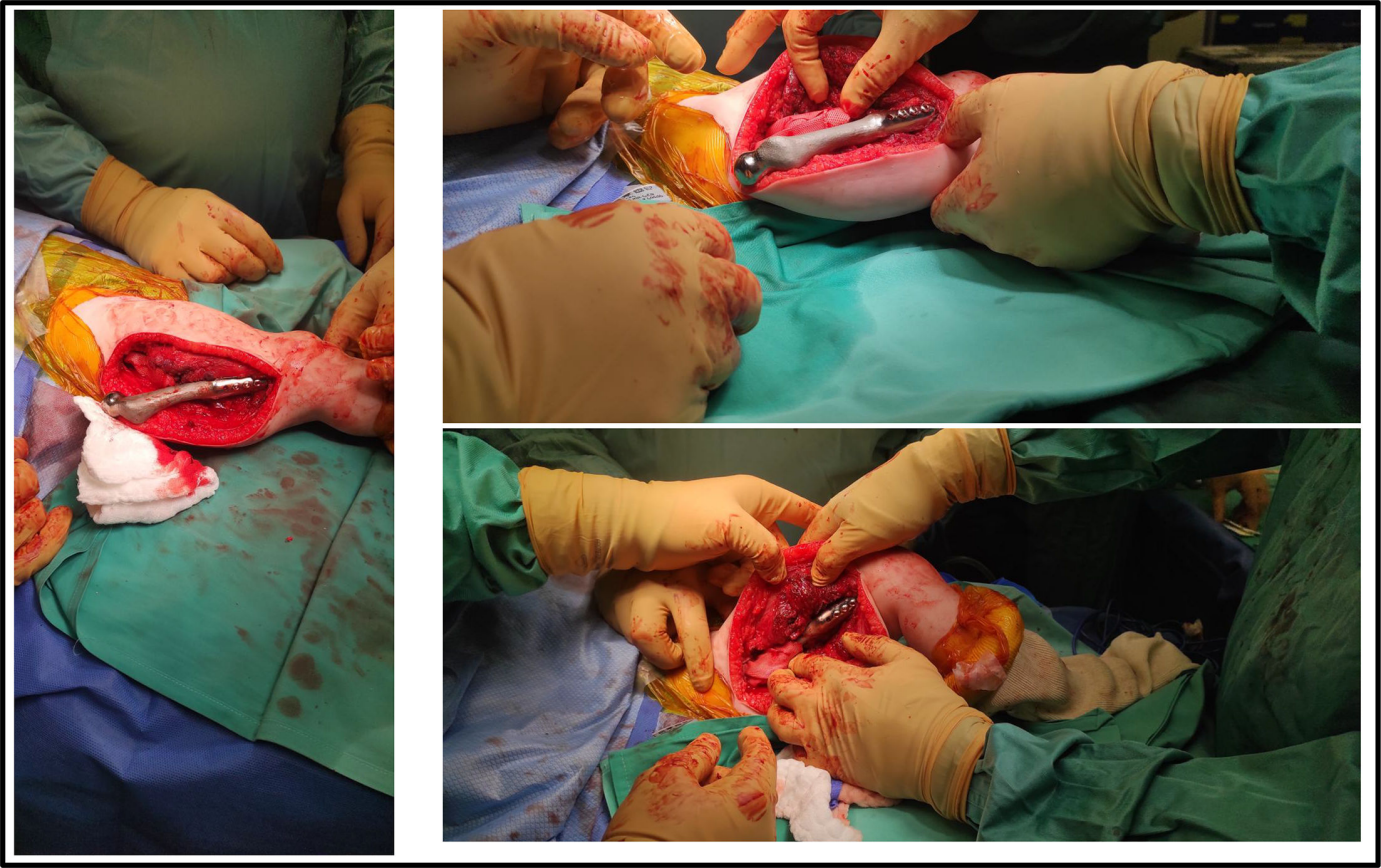
Intraoperative views of a two-year-old female with distal femoral osteosarcoma (patient #21). After oncological resection, the prepared bone surfaces were reconstructed using a custom Ti6Al-4V implant and 1:1 anatomical model verification. The implant achieved accurate alignment and stable seating within the reconstructed femoral site.

Standard surgical techniques were employed for tumour exposure and resection, with the goal of achieving negative surgical margins. Osteotomies were then performed with high precision using the PSIs that had been 3D printed in advance. During the procedure, the custom Ti-6Al-4V implants demonstrated an accurate fit within the multi-planar bone defects created by the resections. Figures 5 to 7 show intraoperative images of three representative patients. In select cases, autologous bone graft harvested from the patient’s pelvis was used to further enhance osseointegration. Postoperative management and follow-up adhered to established limb salvage protocols, with rehabilitation individualized based on factors such as tumour location, extent of bone resection, residual host bone, component size, and overall bone quality. Typically, patients began with a six-week period of non-weightbearing, followed by a gradual return to full weightbearing as tolerated.

**Fig. 8:**
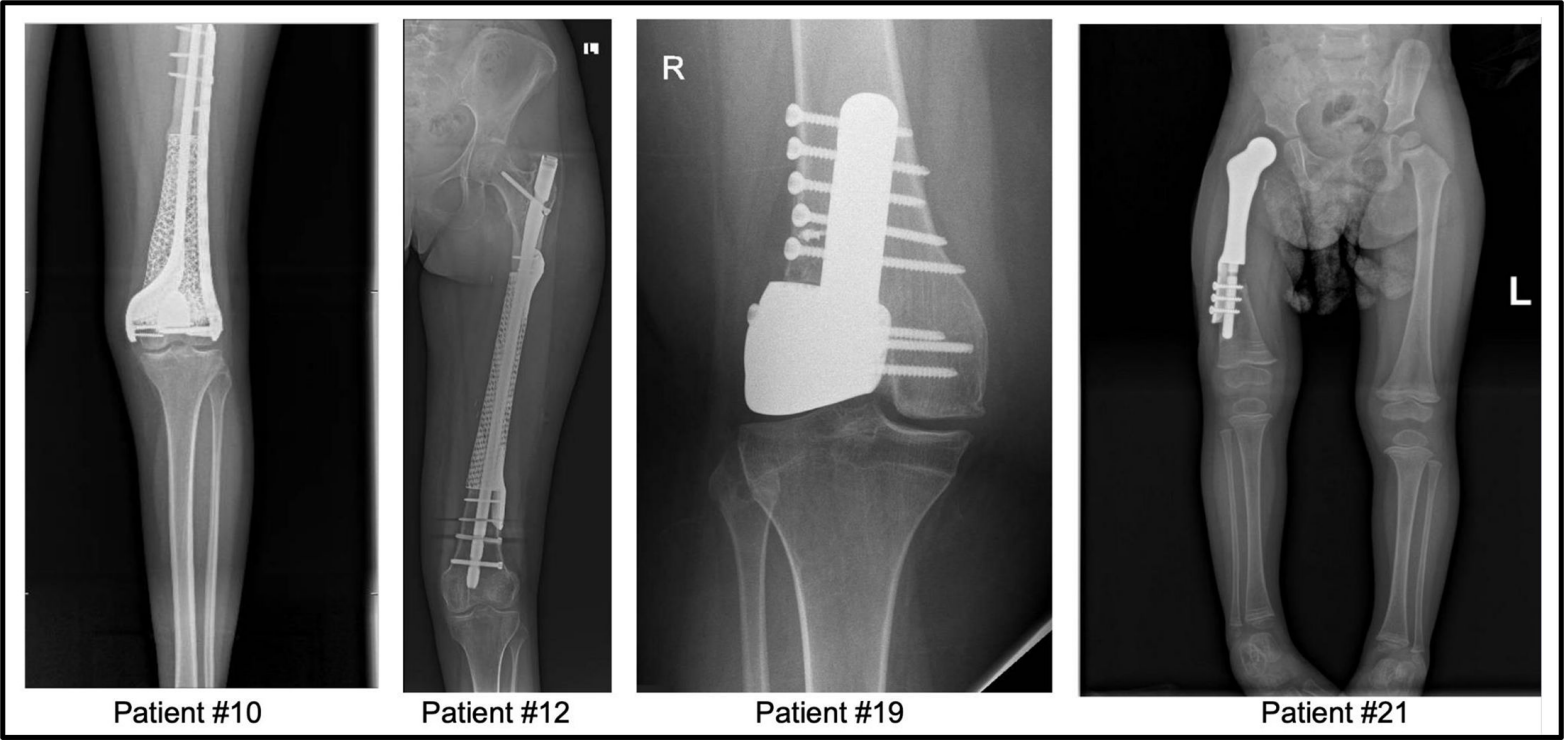
Anteroposterior postoperative radiographs demonstrating reconstruction with patient-specific Ti-6Al-4V lattice implants, from left to right showing: a) Patient #10, a 17-year-old female with distal femoral osteosarcoma reconstructed with a custom lattice implant and distal plate fixation. b) Patient #12, a 14-year-old female with mid-shaft femoral Ewing's sarcoma reconstructed with a custom lattice implant reinforced with an intramedullary nail. c) Patient #19, a 15-year-old male with distal femoral geographical chondroblastoma resection and reconstruction using a custom lattice implant. d) Patient #21, a two-year-old female with distal femoral osteosarcoma showing a custom proximal femur arthroplasty implant.

Radiological assessment confirmed correct positioning and secure fixation of the Ti-6Al-4V implants. Routine postoperative radiographs were used to monitor the outcomes of bone reconstruction and implant stability. Figure 8 shows postoperative radiographs of four representative patients.

## Results

Most reconstructions followed intercalary resection (25/29, 85%), three resections were geographical (3/29, 10%), and one was disarticulation with hip reconstruction (1/29, 3%). The mean surgical resection length from the entire bone length was 36.4 (11.5%). Intraoperative complications were not recorded. Early postoperative complications occurred in three patients (10.3%): one haematoma treated with irrigation and debridement (I&D) at postoperative day (POD) 2, and two deep infections treated with I&D at three and four weeks. Late complications were documented in four patients (13.7%): one infection ultimately required implant removal with free fibula grafting at six months, one mechanical failure was revised to a megaprosthesis at four months, one subtalar fusion was performed for nonunion at four months. Femoral component loosening with femoral hip subluxation was observed in a two-year-old female with osteosarcoma (patient #21). Owing to diffuse metastatic disease and poor response to chemotherapy treatment, the patient was managed with palliative care, and no further surgical intervention was performed. Two amputations occurred during follow-up: one below-knee amputation at 25 months at the patient’s request (no complications were mentioned), and one above-knee amputation for local recurrence, corresponding to a limb-salvage rate of 27/29 (93.1%). Functional recovery, assessed by MSTS-93, was available for 20 patients at the latest follow-up, with a median of 80% (IQR 60% to 87%) (Table II).

**Table II.** Surgical procedure, complications, resection, and Musculoskeletal Tumor Society scores.

Patient #	Resection	Short-term complications	Long-term complications	Resection length, mm\limb length, mm, n (%)	MSTS at final follow-up, %
1	Intercalary	None	None	N/A	87
2	Intercalary	None	None	92/430 (21)	N/A
3	Intercalary	None	None	134/420 (32)	80
4	Intercalary + ankle fusion	None	None (BKA under patient request at 2 years)	131/366 (36)	40
5	Intercalary	None	None	89/383 (23)	N/A
6	Intercalary	None	None	92/391 (24)	N/A
7	Intercalary	None	Revision to Mega prosthesis at 4 months (mechanical failure)	127/354 (36)	10
8	Intercalary	None	None	209/435 (48)	N/A
9	Intercalary	None	None	127/354 (36)	87
10	Intercalary	None	None	170/451 (38)	73
11	Geographical	None	Implant removal at 6 months (infection) + free fibula graft at 15 months	97/379 (26)	87
12	Intercalary	None	None	196/410 (48)	87
13	Intercalary	None	None	N/A	80
14	Intercalary	I&D at POD2 (haematoma)	None	92/392 (23)	80
15	Intercalary	None	None	240/425 (56)	83
16	Geographical	None	None	67/337 (20)	93
17	Intercalary	None	None	270/348 (56)	93
18	Disarticulation	I&D at 1 month (Infection)	None	N/A	60
19	Geographical	None	None	N/A	83
20	Intercalary	None	Subtalar fusion at 4 months (nonunion)	84/369 (23)	43
21	Intercalary	None	Component loosening and femoral hip subluxation	83/157 (53)	77
22	Intercalary	None	None	88/315 (28)	87
23	Intercalary	None	None	N/A	N/A
24	Intercalary	None	None	145/446 (33)	80
25	Intercalary	I&D at POD 21 (Infection)	None	145/377 (38)	50
26	Intercalary	None	None	160/480 (33)	73
27	Intercalary + ankle fusion	None	None	142/329 (43)	57
28	Intercalary	None	None	71/436 (16)	70
29	Intercalary	None	None	21/41 (51)	43

BKA, below-knee amputation; I&D, irrigation and debridement; MSTS, Musculoskeletal Tumor Society Score; N/A, not available; POD, postoperative day.

Regarding oncological parameters, soft-tissue margins were R0 in 13 patients, R1 in five patients, and R2 in one patient; margin data were not reported for the remainder. Importantly, 28/29 patients had negative bone margins; the single R2 case is addressed in the Discussion section. Histological tumour necrosis was available in 16 cases with a mean of 73.7 (SD 28.3%; median 85%). At last follow-up, oncological status across the whole cohort was no evidence of disease in 23 patients, alive with disease in two patients, and dead of disease in three patients; one patient was operated following trauma with no oncological history. Metastasis was recorded in seven patients (most commonly to the lungs), and local recurrence in four patients; one patient developed a secondary acute myeloid leukaemia (Table III).

**Table III.** Enneking classification, margins, necrosis, and oncological status.

Patient #	Enneking	Margins	Necrosis	Oncological event	Oncological status
1	-	-	-	Nononcological	Nononcological
2	2a	-	-	Metastasis (lung)	DOD
3	Benign	-	-	None	NED
4	2b	-	-	None	NED
5	2b	R0	95	Metastasis (abdomen & thorax) + LR (AKA due to LR)	DOD
6	2b	R0	90	Metastasis (lung) + LR	DOD
7	2b	R2	80	Secondary AML	NED
8	2b	R0	92	Metastasis (sternum + lung) + LR	NED
9	2b	-	-	None	NED
10	2b	R0	100	None	NED
11	2b	R0	40	None	NED
12	2b	R0	50	None	NED
13	1b	R1	-	None	NED
14	2b	R0	99	Metastasis (lung + perirenal fat)	AED
15	3	R0	93	Metastasis (lung)	NED
16	1b	-	-	None	NED
17	1a	R0	-	None	NED
18	2b	-	75	None	NED
19	Benign	R1	-	None	NED
20	1b	R1	-	None	NED
21	2b	R0	60	Metastasis (lung + left pelvic)	AED
22	2b	R0	95	None	NED
23	2a	R0	-	None	NED
24	2b	R1	40	None	NED
25	2b	-	-	None	NED
26	2b	R0	80	None	NED
27	1b	R1	90	None	NED
28	1a	-	-	None	NED
29	2b	-	-	None	NED

AED, alive with evidence of disease; AKA, above-knee amputation; AML, acute myeloid leukemia; DOD, dead of disease; LR, local recurrence ; NED, no evidence of disease .

## Discussion

In this study, we present the clinical results of a novel workflow for reconstruction of critical-sized bone defects following weightbearing long-bone tumour resections. This work follows our previous study,^15^ which detailed the technical and mechanical characteristics of these implants. We report on 28 patients with primary bone tumour resections guided by intraoperative 3D-printed osteotomy PSIs, and one patient with nonunion following traumatic injury. All patients underwent bone reconstruction using patient-specific Ti-6Al-4V implants. A regenerative approach was adopted, designing the implant with a lattice body that serves as a scaffold for biological bone filling and reinforcing it with orthopaedic fixation for mechanical strength and stability. The multidisciplinary workflow enhanced the specificity and accuracy of the 3D-printed parts, improving implant design and surgical planning. The workflow continues to evolve as experience grows.

In recent years, customized AM titanium implants have gained popularity in orthopaedic oncology, particularly for load-bearing bones.^21^ This is due to their superior fit in complex tumour resections, revisions, or deformities, and in tumours near joints.^22^ Although evidence-based studies are limited, especially regarding long-term outcomes, promising results have been reported.^5^ Attempts to reconstruct long-bone defects using Ti-6Al-4V implants as fully bulked constructs^22-25^ with porous edges,^2,6,11,18^ or mesh/porous segments integrated with intramedullary devices,^26-28^ have shown good short-term outcomes, though some cases experienced aseptic loosening or implant fracture requiring revision. Our findings demonstrate that patient-specific Ti-6Al-4V implants for critical-sized bone defect reconstruction can achieve functional recovery and accurate anatomical repair without compromising surgical or oncological outcomes. All implants incorporated a lattice body reinforced with orthopaedic fixation (e.g. intramedullary nail, component, and side plates). The lattice promotes bone formation within and over its surface, creating a gradual transition between native bone and metallic struts that mitigates fracture risk at the interface.

This cohort shows that patient-specific 3D-printed Ti-6Al-4V implants function as a versatile limb-salvage method across a genuinely mixed cohort. Each patient was followed for at least 12 months evaluating surgical, functional, and oncological outcomes. Early surgical morbidity rates were low as well as mechanical failure rates, with fewer revision surgeries necessary as compared with standard implants in the field of orthopaedic oncology.^29^ Local oncological control with a negative margin resection was achieved in 96% of the cases. Owing to a technical error, the PSI in our only R2 resection did not achieve an anatomical fit intraoperatively, resulting in a positive resection margin. The same patient subsequently experienced implant mechanical failure. At that stage, we had not yet instituted routine prospective finite element analysis; a retrospective FEA performed post hoc localized peak stresses at the eventual failure site (see our previous study for additional details^15^). We treated this as a sentinel case, and it illustrates our learning curve. Subsequently we implemented prospective FEA for all patients, and improved PSI design and intraoperative verification. Prospectively, no further PSI misfits or similar failures have occurred in our cohort. Most patients (27/29, 93%) achieved limb salvage surgery. One of these patients requested a below-knee amputation (BKA) due to individual preferences. This 27-year-old, physically active patient suffered from Ewing's sarcoma at the distal tibia. The operation required an intercalary resection with an implant reconstruction in addition to ankle fusion. He had no complications, yet he felt debilitated and could not do sport activities as he wished. After several discussion he decided to undergo a BKA, and he was happy with the final functional results allowing him to return to sports. The second patient was a 44-year-old male with malignant fibrous histiocytoma (MFH) of the tibia who experienced a disease flare with both metastatic progression and local recurrence. The local recurrence was managed with an above-knee amputation to achieve negative margins; unfortunately, he subsequently died of disease.

Several limitations apply to patient-specific Ti-6Al-4V implants in general, and to our study in particular. Because this is a relatively new approach to implant design and manufacturing, further research is needed to define optimal materials and their characteristics (e.g. mechanical properties, surface treatments) and to establish standards that support wider clinical adoption. In addition, the cost and cost-effectiveness of patient-specific Ti-6Al-4V implants, including manufacturing, planning, and peri-operative resource use, must be evaluated alongside clinical outcomes to inform broader implementation decisions. The advantages reported here and elsewhere mainly stem from small cohorts or single-case reports, and the lack of control groups limits objective comparisons. Our follow-up period of a minimum of one year is suitable for assessing early stability and function, but insufficient to fully understand late complications or long-term durability. Notably, our cohort is heterogeneous, spanning paediatric to older adult patients, oncological and non-oncological indications, tibial and femoral sites, and various resection types. Additionally, adjuvant treatments and rehabilitation protocols varied across cases. This diversity enhances external generalizability but also introduces confounding factors and increases variability in functional and complication outcomes; learning-curve effects throughout the study period may further bias results towards later cases. Larger, controlled, and ideally multicentre studies with longer follow-up, standardized rehabilitation and outcome measures, and predefined design and manufacturing protocols are necessary to confirm and optimize the clinical benefits of customized Ti-6Al-4V lattice implants.

In conclusion, in this study, we demonstrated that patient-specific Ti-6Al-4V lattice implants reinforced with orthopaedic fixation provide an effective limb-salvage solution for critical-sized, weightbearing femoral and tibial defects. The constructs offered stable structural restoration, acceptable complication rates, and oncological outcomes consistent with current standards, enabling progressive functional recovery. This workflow supports not only limb salvage, but also, when feasible, joint preservation, and can be extended to complex reconstructions such as severe trauma where conventional implants are inadequate. Continued clinical validation with larger cohorts and longer follow-up, alongside optimization of lattice design for mechanical strength and osseointegration, will help establish this approach as a clinical standard for patient-specific reconstruction of critical-sized bone defects.

**Take home message**

- Patient-specific titanium implants are transforming the reconstruction of critical-sized, weightbearing bone defects after oncological resection.

## Data Availability

The data that support the findings for this study are available to other researchers from the corresponding author upon reasonable request.
